# Response to PD-1 inhibitor in SMARCB1‑deficient undifferentiated rectal carcinoma with low TMB, proficient MMR and BRAF V600E mutation: a case report and literature review

**DOI:** 10.1186/s13000-023-01415-8

**Published:** 2024-01-12

**Authors:** Wenjuan Shen, Yi Pan, Shuangmei Zou

**Affiliations:** Department of Pathology, National Cancer Center/National Clinical Research Center for Cancer/Cancer Hospital, Chinese Academy of Medical Sciences and Peking Union Medical College, Beijing, 100021 People’s Republic of China

**Keywords:** Response, PD-L1, SMARCB1, Rectal carcinoma, Case report

## Abstract

**Background:**

Despite major advancements, effective treatment for patients with SMARCB1-deficient cancers has remained elusive. Here, we report the first case of a SMARCB1-deficient undifferentiated carcinoma in the rectum expressing high PD-L1 and responding to a PD-1 inhibitor, as well as with low tumor mutation burden (TMB), proficient mismatch repair (MMR) and BRAF V600E mutation.

**Case presentation:**

A 35-year-old man visited our hospital complaining of increased defecation frequency, bloody stools and weight loss of 3 kg for one month. Colonoscopy revealed an ulcerated and irregular mass approximately 8–12 cm from the anus. Surgical resection was performed. Histopathological findings revealed that the tumor cells had poor connectivity with each other; each cell had eosinophilic cytoplasm and a polymorphic nucleus. Brisk mitotic activity and necrosis were frequently observed in the tumor cells. Immunohistochemical examination showed that the tumor cells were negative for SMARCB1. The tumor proportion score (TPS) of PD-L1 (22C3) expression was 95%, and the combined positive score (CPS) was 100; the tumor was mismatch repair (MMR) proficient. Next-generation sequencing showed a low tumor mutation burden (TMB), as well as the BRAF V600E mutation. The final diagnosis was SMARCB1-deficient undifferentiated carcinoma. Chemotherapy was useless in this case. His tumor recurred during chemotherapy, and he then received targeted therapy with tirelizumab, an inhibitor of PD-1. At present, his general condition is good. A recent computed tomography (CT) scan showed that the tumor had disappeared, indicating that the immunotherapy was effective. Astonishingly, his most recent follow-up was in August, and his condition continued to improve with the tumor has disappeared.

**Conclusion:**

SMARCB1‑deficient undifferentiated carcinoma in the rectum is extremely rare, and it has aggressive histological malignancy and poor progression. The observed response to PD-1 inhibitors suggests a role for prospective use of SMARCB1 alterations as a predictive marker for immune checkpoint blockade.

**Supplementary Information:**

The online version contains supplementary material available at 10.1186/s13000-023-01415-8.

## Background

SMARCB1 (SWI/SNF-related matrix-associated act independent regulator of chromatin subfamily B member 1, also known as INI-1 and BAF47) is a tumor-suppressor gene located on chromosome 22q11.2 [1, 2]. SMARCB1 is part of the SWI/SNF chromatin remodeling complex, which plays a role in transcriptional regulation. Loss of SMARCB1 was originally described in association with malignant rhabdoid tumors in the pediatric population [3, 4]. Modern advances in diagnostic techniques have occasionally identified SMARCB1 loss in tumors from multiple sites [5–10], broadly referred to as SMARCB1-deficient cancers. Current estimates indicate that 1.4% of all cancers contain SMARCB1 alterations (1152 of 84,646 queried samples found in AACR Project GENIE Cohort v11.0) [11]. Despite these major advancements, effective treatment for patients with SMARCB1-deficient cancers has remained elusive. It is important to utilize gains in our understanding to advance potential therapeutic treatments. Here, we report the first case of a SMARCB1-deficient undifferentiated carcinoma expressing high PD-L1 and responding to a PD-1 inhibitor, with low tumor mutation burden (TMB), proficient mismatch repair (MMR) and BRAF V600E mutation in the rectum. Moreover, we discuss the role of SMARCB1 alterations in diagnosis and treatment and predictive markers of potential targeted therapy or immunotherapy of colorectal cancer (CRC).

## Case presentation

**Fig. 1:**
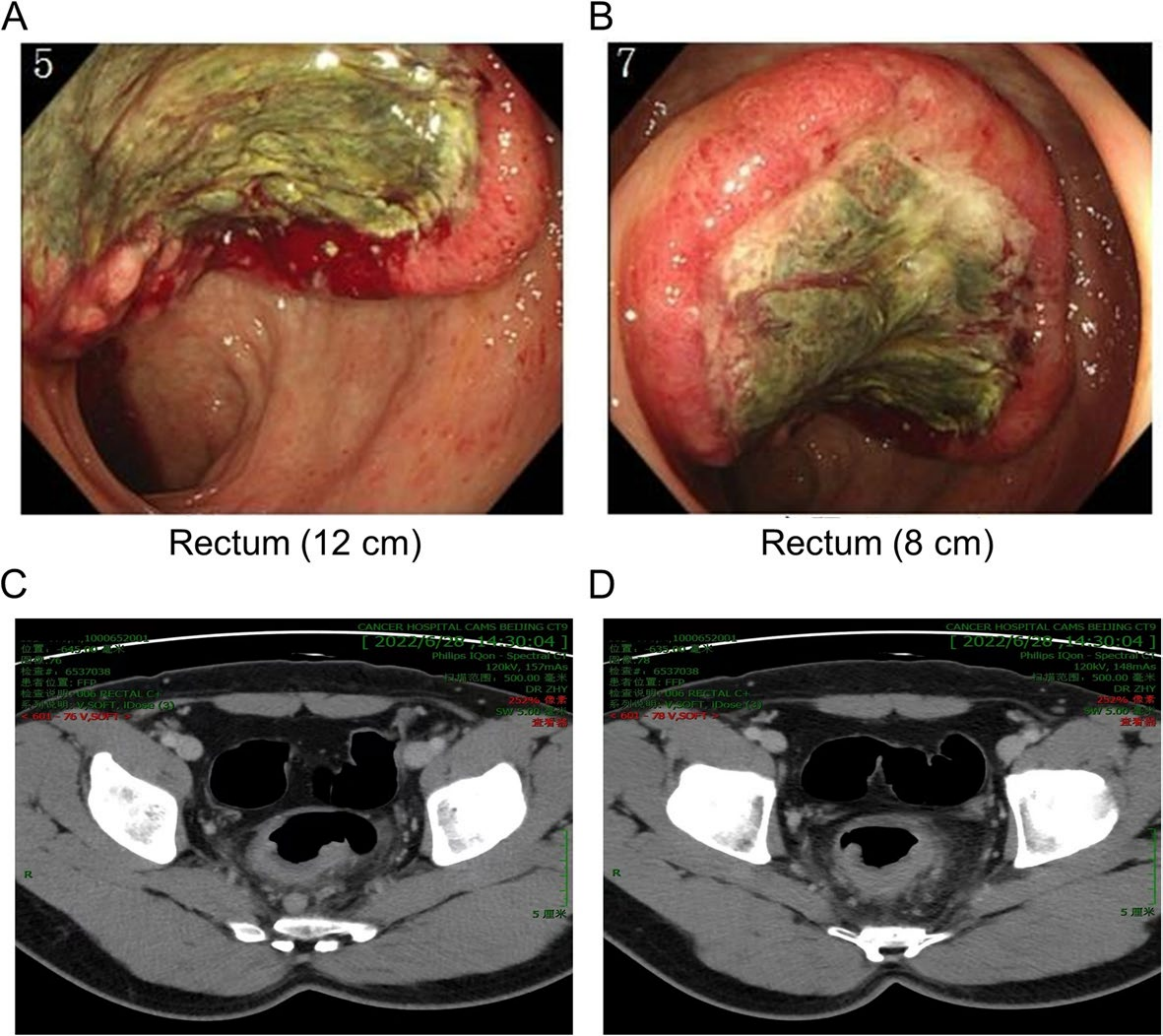
Clinical findings. **A**, **B** Colonoscopy revealed an ulcerated and irregular mass approximately 8-12 cm from the anus with obvious lumen stenosis. **C**, **D** Computed tomography (CT) shows thickened walls of the sigmoid and upper rectum. Multiple regional lymph nodes showed strengthening signals

A 35-year-old man visited our hospital complaining of increased defecation frequency, bloody stools and weight loss of 3 kg for one month. The patient denied any history of malignant tumors. And the patient also denied any family history of malignant tumors. Physical rectal examination revealed a circumferential mass with poor activity. Blood was noted by digital rectal exam. Laboratory findings showed that the tumor markers carcinoembryonic antigen (CEA) and carbohydrate antigen 19-9 (CA19-9) were within the normal range. Colonoscopy suggested an ulcerated and irregular mass approximately 8-12 cm from the anus with obvious lumen stenosis (Fig. 1A and B). The biopsy result of the rectum lesion indicated poorly differentiated adenocarcinoma. A computed tomography (CT) scan showed a wall-thickening lesion in the sigmoid colon and upper rectum (Fig. 1C and D). Multiple regional lymph nodes showed strengthening signals. The lung and liver were unremarkable.

**Fig. 2:**
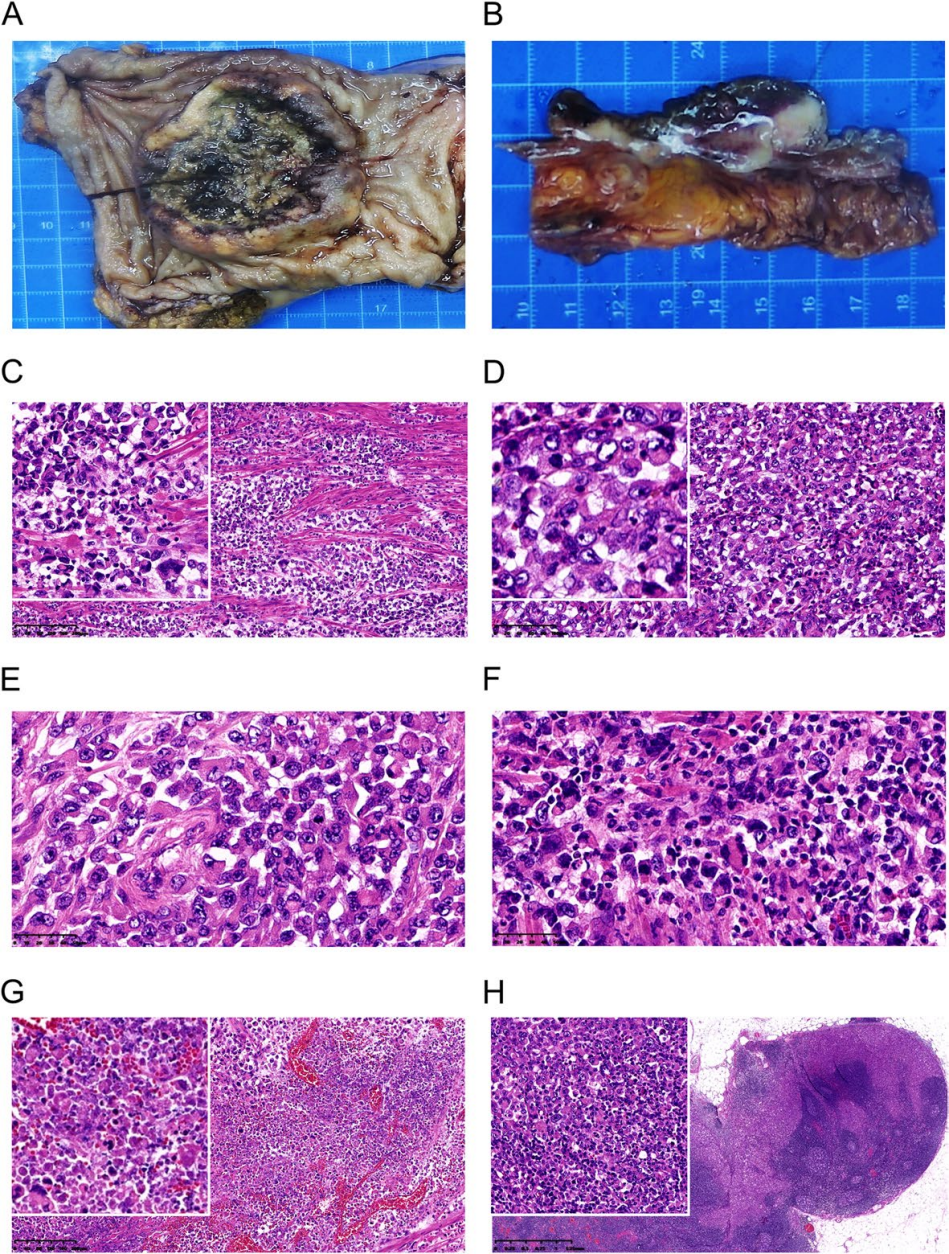
Pathological findings. **A**, **B** The tumor, measuring 6.5 cm × 6.2 cm × 1.5 cm, occupied 3/4 of the rectal lumen. It presented as a well-demarcated mass lesion with central deep ulceration. The cut surface showed a grayish-white or grayish-yellow solid tumor with central hemorrhage and necrosis. The tumor had invaded through the muscularis propria into the peri-rectal tissues. **C**-**G** Histopathological findings revealed that the tumor cells had poor connectivity, eosinophilic cytoplasm and a polymorphic nucleus. **H** Metastasis was observed in regional lymph nodes

The surgical specimen consisted of a sigmoid and rectal segment measuring approximately 15 cm in length, with a luminal diameter varying from approximately 5 cm at the proximal end to 9.0 cm at the distal end. The tumor, measuring 6.5 cm×6.2 cm×1.5 cm, occupied 3/4 of the rectal lumen. It presented as a well-demarcated mass lesion with central deep ulceration (Fig. 2A). The cut surface showed a grayish-white or grayish-yellow solid tumor with central hemorrhage and necrosis. The tumor had invaded through the muscularis propria into the peri-rectal tissues (Fig. 2B). Forty-six lymph nodes were retrieved from the adipose tissue surrounding the bowel wall, with a diameter of 0.3 cm (4.5×3.5×1.5 cm).

Histopathological findings revealed that the tumor cells had poor connectivity, eosinophilic cytoplasm and a polymorphic nucleus (Fig. 2C). These characteristics are called rhabdoid features because the morphology of these cells is similar to that of rhabdomyosarcoma tumor cells. These rhabdoid cells contained vesicular nuclei with prominent nucleoli and a significant amount of eosinophilic cytoplasm (Fig. 2D and E). Brisk mitotic activity was a frequent finding (Fig. 2E and F). The remaining 35% of the maximal cut surface was not evaluated due to necrosis (Fig. 2G). The tumor had invaded the peri-rectal tissues and showed lymph-vascular and perineural invasion. The surgical margins were tumor free. Metastasis was observed in 12 of the 46 regional lymph nodes retrieved (Fig. 2H).

**Fig. 3:**
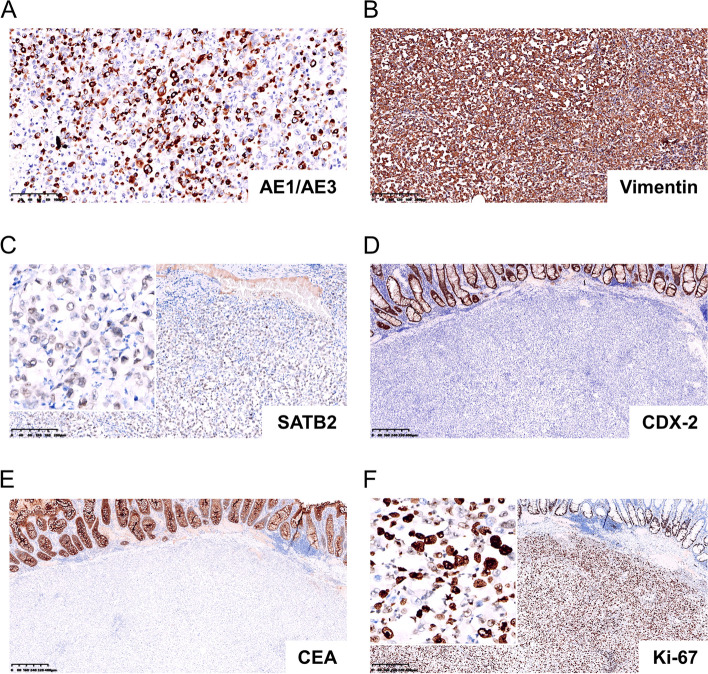
Immunohistochemistry results of tumor cells. The tumor cells were positive for cytokeratin AE1/AE3 (**A**) and vimentin (**B**). The tumor cells showed weak positivity for SATB2 (**C**). CDX-2 (**D**) and CEA (**E**) were negative. Ki-67(**F**) was as high as 95%

**Fig. 4:**
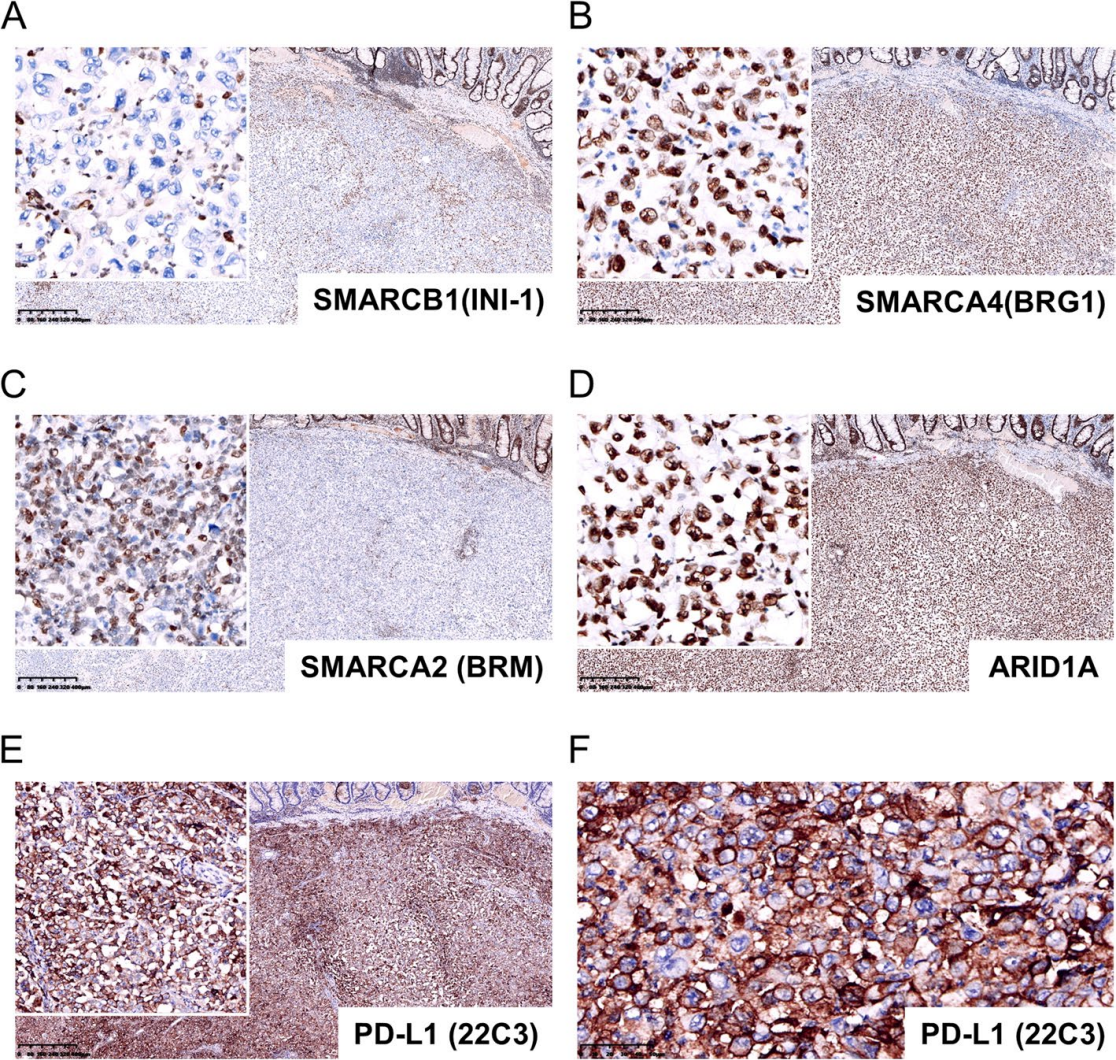
Immunohistochemistry results of tumor cells. **A** The tumor cells showed complete loss of INI-1 (SMARCB1 protein). **B**-**D **The tumor cells were diffusely positive for BRG1 (SMARCA4), BRM (SMARCA2) and ARID1A. **E** and **F** The tumor proportion score (TPS) of PD-L1 (22C3) expression was 95%, and the combined positive score (CPS) was over 100

**Fig. 5:**
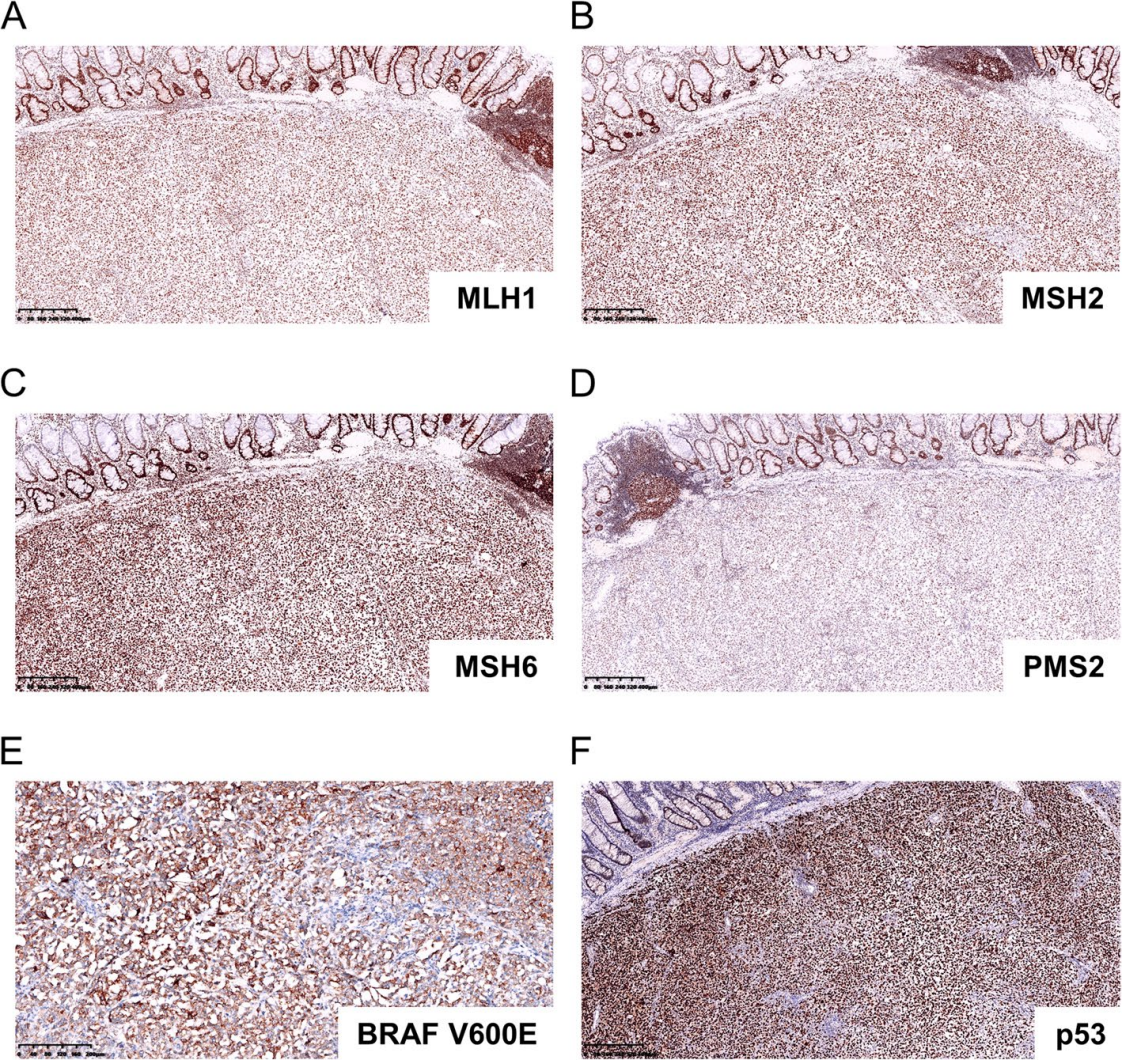
Immunohistochemistry for therapeutic markers in tumor cells. **A**-**D ** MLH1, MSH2, MSH6 and PMS2 indicated proficient MMR. **E** and **F ** BRAF V600E and p53 mutant protein expression were strong and diffuse

Immunohistochemistry results showed that the tumor cells were positive for both epithelial (cytokeratin AE1/AE3, Fig. 3A) and mesenchymal (Vimentin, Fig. 3B) cell markers. Tumor cells showed weak positivity for SATB2 (Fig. 3C). However, CDX-2 (Fig. 3D) and CEA (Fig. 3E), which are markers of colonocyte differentiation, were negative. Ki-67, which shows proliferative ability, was as high as 95% (Fig. 3F). Notably, the tumor cells showed complete loss of INI-1 (SMARCB1 protein), which is normally expressed in the nucleus (Fig. 4A). Nuclear expression was retained in neighboring nonneoplastic cells. They were diffusely positive for BRG1 (SMARCA4, Fig. 4B), BRM (SMARCA2, Fig. 4C) and ARID1A1 (Fig. 4D). Impressively, the tumor proportion score (TPS) of PD-L1 (22C3) expression was 95%, and the combined positive score (CPS) was 100 (Fig. 4E and F). However, probing for MMR proteins, including MLH1 (Fig. 5A), MSH2 (Fig. 5B), MSH6 (Fig. 5C) and PMS2 (Fig. 5D), revealed proficient MMR, suggesting low microsatellite instability (low-MSI) or microsatellite stability (MSS). Additional immunohistochemical staining for BRAF V600E (Fig. 5E) and p53 (Fig. 5F) mutant proteins indicated strong and diffuse expression. The tumor cells were negative for CK7, CK20, CD56, ChrA, Desmin, MyoD1, Melan A, S-100, HER-2 and LCA (Supplementary Fig. 1).

Next-generation sequencing was also performed, and the tumor exhibited a tumor mutation burden (TMB) of 5.84 mutations/megabase (Supplementary Fig. 2A) and microsatellite stability (MSS, Supplementary Fig. 2B). The results also indicated the presence of BRAF (c.1799T>A, p.V600E) mutation and TP53 (c.818 G>A, p.R273H) mutation.

**Fig. 6:**
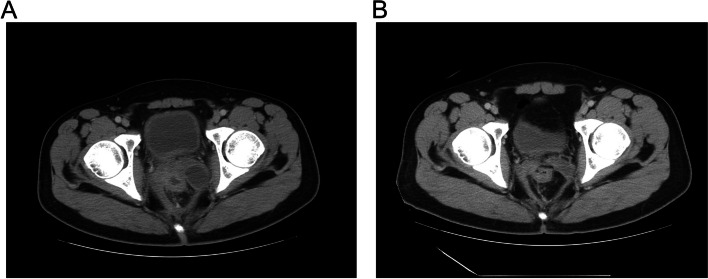
Follow-up condition of the patient. **A** The tumor recurred during chemotherapy. **B** The recent computed tomography (CT) scan showed that the tumor had disappeared after the patient received 4 cycles of immunotherapy

The final pathological diagnosis was SMARCB1‑deficient undifferentiated carcinoma, pT3N2bM0 (Stage IIIc), according to the World Health Organization classification of digestive system tumors, 5th edition. Postoperative adjuvant chemotherapy was administered using XELOX (capecitabine oxaliplatin). Unfortunately, the tumor recurred while the patient was receiving chemotherapy (Fig. 6A). Then, he received targeted therapy with tirelizumab, a monoclonal antibody against PD-1. At present, he has received 4 cycles of immunotherapy, and his general condition is good. A computed tomography (CT) scan showed that the tumor had disappeared (Fig. 6B), indicating that the above immunotherapy was effective. At nearly one year postoperatively, the patient was still alive, and his general condition was good. Astonishingly, his most recent follow-up was in August, and his condition continued to improve with the tumor has disappeared.

## Discussion

We describe an uncommon case of a middle-aged man presenting with SMARCB1-deficient undifferentiated carcinoma. To the best of our knowledge, this is the first case report of SMARCB1-deficient undifferentiated carcinoma in the rectum expressing high PD-L1 and responding to PD-1 inhibitors, as well as with low tumor mutation burden (TMB), proficient mismatch repair (MMR) and BRAF V600E mutation.

The SWI/SNF complex functions in chromosome modeling and regulates gene transcription, cell proliferation, lineage-specific differentiation, and DNA damage repair. Inactivating mutations in the subunits can lead to deficiency of the SWI/SNF complex and thus influence cell differentiation; it is usually associated with undifferentiated histological morphology and highly aggressive clinical behavior. Villatoro et al. [12] found that 7% of 338 colorectal adenocarcinoma cases have deficient SWI/SNF expression. Recently, Ahadi et al. [10] reported loss of SMARCA4 expression in 13 (0.3%), loss of SMARCA2 expression in 59 (1.3%), and loss of SMARCB1 expression in 21 (0.4%) among 4508 consecutive resected CRC cases. SWI/SNF complex deficiency is associated with higher grade, a right-sided location, mismatch repair deficiency, and BRAF V600E mutation (*P* < 0.05) [10]. ARID1A expression is most studied in colorectal carcinoma. ARID1A-deficient expression in colorectal carcinoma is frequently associated with MMR protein deficiency and BRAF mutation and exhibits medullary differentiation and mucinous differentiation. In contrast, colorectal carcinoma with SMARCA2-deficient expression displays conventional gland-forming histologic features and is less likely to exhibit MMR deficiency [12].

Immune checkpoint blockade for cancer therapy with anti-programmed death ligand 1 (anti-PD-L1) antibodies has been approved, highlighting a dynamic change in the field of immuno-oncology. Some established biomarkers include deficient MMR/microsatellite instability-high (MSI-H) [10, 13] and high TMB (> 10 mutations/megabase) [14, 15]. Metastatic colorectal carcinoma patients with deficient MMR/MSI-H have a significant survival benefit from immune checkpoint inhibitor therapy [16]. Importantly, this SMARCB1-deficient rectal undifferentiated carcinoma showed low TMB (only 5.84 mutation/megabase) and proficient MMR but surprisingly had significant PD-L1 expression. Although it was not possible to assess prognosis in one patient, SMARCB1 may also predict immune checkpoint blockade responsiveness independently, which supports the notion that SMARCB1 deficiency may represent a primary event rather than a secondary phenomenon in MSI-associated carcinoma. Tumor immunity may be affected in multiple ways. SMARCB1‑deficient tumors show infiltration by subpopulations of clonally expanded T cells, suggesting a tumor-specific immune response [17]. Case reports of 3 patients with SMARCB1-loss aggressive pediatric cancers demonstrate evidence of response to immune checkpoint blockade [18]. In one study, an adult patient with recurrent SMARCB1-loss renal medullary carcinoma had complete response to nivolumab lasting greater than 9 months despite low TMB [6]. Another patient with advanced refractory, SMARCB1-deficient epithelioid sarcoma achieved complete remission with combined ipilimumab and nivolumab [19]. Much of these data have only recently emerged, and some of the clinical links between SMARCB1 loss and immunotherapy remain anecdotal or a matter of debate [20]. Nonetheless, these links merit further understanding and exploration because of their high potential impact in the patient care setting.

In BRAF, a missense mutation (V600E) of exon 15 codon 600 activates downstream signaling [21]. Only approximately 5.6% of colorectal cancers harbor this mutation, and prognosis in these cases is known to be poor in Asian individuals [22]. Regarding undifferentiated colorectal cancer with SMARCB1-deficient features, the BRAF V600VE mutation was observed in 76.2% (16/21) of reported CRC cases [10]. We hypothesize some correlation between BRAF mutation and SMARCB1-defcient features in colorectal cancers. Elucidation of the molecular mechanism is warranted. On the other hand, BRAF-mutant colorectal cancer is reported to be common in sporadic MSI-high right-sided colon cancers [23]. Our case involved proficient MMR in a rectal tumor, but one case is not sufficient to make any conclusion. Along with the accumulation of cases, elucidation of the correlation between MSI status and SMARCB1-deficient features should be addressed in the future.

Forrest SJ et al. found many INI-1-negative tumors express PD-L1, suggest that clinical trials of immune checkpoint inhibitors are warranted in INI-1-negative pediatric cancers [24]. We all know that the SWI/SNF family of chromatin remodeling complexes include ARID1B, ARID1A, SMARCB1, SMARCA4, SMARCA2 and PBRM1, etc. A study found a higher mutational burden in ARID1A-mutant CRCs, and ARID1A loss was correlated with high PD-L1 expression in stromal cells regardless of MSI status [25], and another study suggested that even ARID1A mutant samples without MSI-high status were TMB-high, had high levels of PD-L1 expression and high estimated infiltrating CTLs [26]. Therefore, we assumed that the loss of SWI/SNF leads to high expression of PD-L1, which responds well to immunotherapy. Our hypothesis was validated in non small cell lung cancer (NSCLC). Naito T et al. [27], their current results suggested that loss of SWI/SNF expression in NSCLC is associated with aggressive clinicopathological features, PD-L1-positive status and high TMB. Zhu G et al. [28], also found that patients with NSCLC who have mutation of the SWI/SNF complex were more likely to benefit from immune checkpoint inhibitors therapy.

Overall, we propose that PD-L1, BRAF V600E, and MMR protein immunohistochemical staining should be performed for all SWI/SNF-deficient undifferentiated carcinomas. However, it remains necessary to assess more cases with detailed information on clinical treatment and prognosis and then to determine if these cases require different treatment strategies, particularly for advanced or recurrent cases.

## Conclusion

SMARCB1-deficient undifferentiated carcinoma in the rectum is extremely rare, and it has aggressive histological malignancy and poor progression. The response to PD-1 inhibitors of the tumor described herein suggests a role for prospective use of SMARCB1 alterations as a predictive marker for immune checkpoint blockade. Further prospective clinical trials of immunotherapy in patients with SWI/SNF complex aberrations and malignancies are urgently needed.

### Supplementary Information

**Additional file 1: Figure 1. **The tumor cells were negative for CK7, CK20, CD56, ChrA, Desmin, MyoD1, Melan A, S-100, HER-2 and LCA.

**Additional file 2: Figure 2. **Next-generation sequencing showed a tumor mutation burden (TMB) of 5.84 mutations/megabase (A) and microsatellite stability (B).

## Data Availability

Not applicable.

## Abbreviations

SMARCB1: SWI/SNF-related matrix-associated act independent regulator of chromatin subfamily B member 1
CRC: Colorectal cancer
MMR: Mismatch repair
MSI: Microsatellite instability
MSS: Microsatellite stability
TMB: Tumor mutation burden
PD-L1: Programmed death ligand 1
TPS: Tumor proportion score
CPS: Combined positive score
