# Supplementary figures and images for: Response to PD-1 inhibitor in SMARCB1‑deficient undifferentiated rectal carcinoma with low TMB, proficient MMR and BRAF V600E mutation: a case report and literature review
Source: Diagn Pathol. 2024 Jan 12;19:11. doi: 10.1186/s13000-023-01415-8 (PMC10785529; doi:10.1186/s13000-023-01415-8)

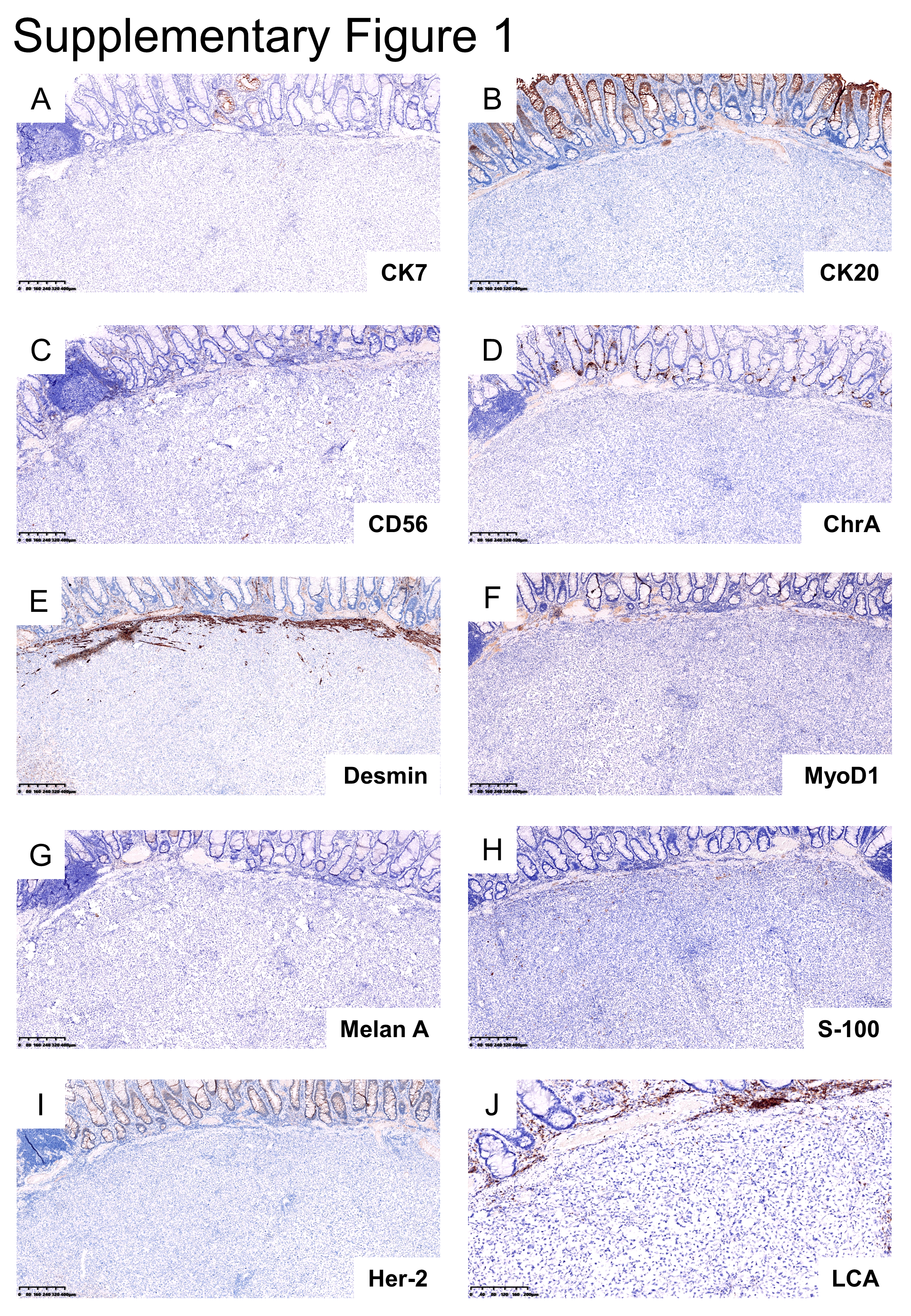

Supplement: Supplementary file 1 — Additional file 1: Figure 1. The tumor cells were negative for CK7, CK20, CD56, ChrA, Desmin, MyoD1, Melan A, S-100, HER-2 and LCA. [file 13000_2023_1415_MOESM1_ESM.tif]

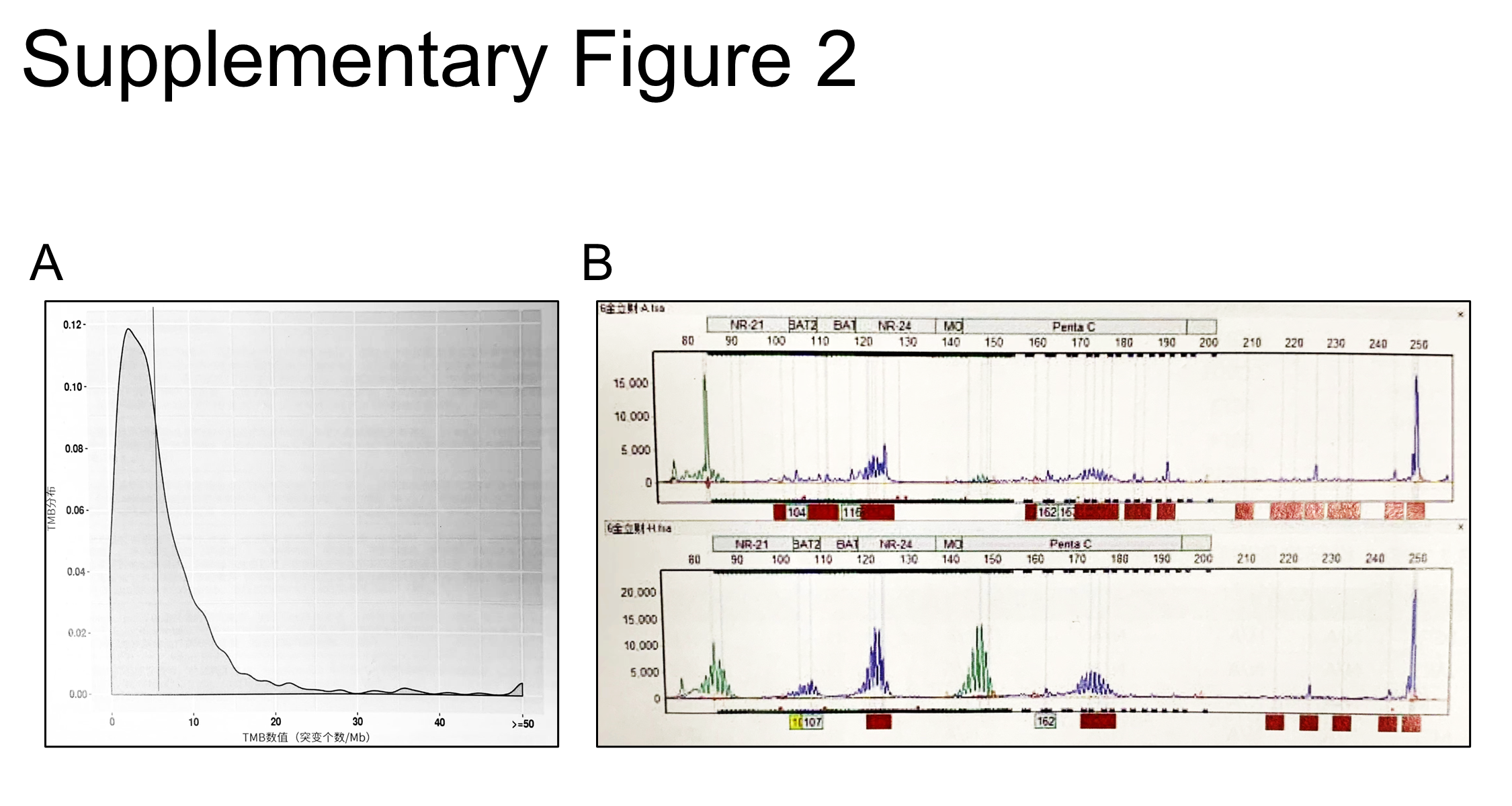

Supplement: Supplementary file 2 — Additional file 2: Figure 2. Next-generation sequencing showed a tumor mutation burden (TMB) of 5.84 mutations/megabase (A) and microsatellite stability (B). [file 13000_2023_1415_MOESM2_ESM.tif]
